# Impaired HDL Function Amplifies Systemic Inflammation in Common Variable Immunodeficiency

**DOI:** 10.1038/s41598-019-45861-1

**Published:** 2019-07-01

**Authors:** Magnhild E. Macpherson, Bente Halvorsen, Arne Yndestad, Thor Ueland, Tom E. Mollnes, Rolf K. Berge, Azita Rashidi, Kari Otterdal, Ida Gregersen, Xiang Y. Kong, Kirsten B. Holven, Pål Aukrust, Børre Fevang, Silje F. Jørgensen

**Affiliations:** 1 Research Institute of Internal Medicine, Division of Surgery, Inflammatory Diseases and Transplantation, Oslo University Hospital, Rikshospitalet, Oslo, Norway; 2 Section of Clinical Immunology and Infectious Diseases, Department of Rheumatology, Dermatology and Infectious Diseases, Oslo University Hospital, Rikshospitalet, Oslo, Norway; 3 Institute of Clinical Medicine, University of Oslo, Oslo, Norway; 4 Department of Clinical Science, University of Bergen, Bergen, Norway; 5 Department of Heart Disease, Haukeland University Hospital, Bergen, Norway; 6 Department of Immunology, Oslo University Hospital, Rikshospitalet, Oslo, Norway; 7 Research Laboratory, Nordland Hospital, Bodø, Norway; 8 Faculty of Health Sciences and K.G. Jebsen TREC, University of Tromsø, Tromsø, Norway; 9 Centre of Molecular Inflammation Research, Norwegian University of Science and Technology, Trondheim, Norway; 10 Department of Nutrition, Institute for Basic Medical Sciences, University of Oslo, Oslo, Norway; 11 Norwegian National Advisory Unit on Familial Hypercholesterolemia, Oslo University Hospital Rikshospitalet, Oslo, Norway

**Keywords:** Primary immunodeficiency disorders, Dyslipidaemias, Molecular medicine

## Abstract

Common variable immunodeficiency (CVID) is the most common symptomatic primary immunodeficiency, characterized by inadequate antibody responses and recurrent bacterial infections. Paradoxically, a majority of CVID patients have non-infectious inflammatory and autoimmune complications, associated with systemic immune activation. Our aim was to explore if HDL, known to have anti-inflammatory properties, had impaired function in CVID patients and thereby contributed to their inflammatory phenotype. We found reduced HDL cholesterol levels in plasma of CVID patients compared to healthy controls, particularly in patients with inflammatory and autoimmune complications, correlating negatively with inflammatory markers CRP and sCD25. Reverse cholesterol transport capacity testing showed reduced serum acceptance capacity for cholesterol in CVID patients with inflammatory and autoimmune complications. They also had reduced cholesterol efflux capacity from macrophages to serum and decreased expression of ATP-binding cassette transporter ABCA1. Human HDL suppressed TLR2-induced TNF release less in blood mononuclear cells from CVID patients, associated with decreased expression of transcriptional factor ATF3. Our data suggest a link between impaired HDL function and systemic inflammation in CVID patients, particularly in those with autoimmune and inflammatory complications. This identifies HDL as a novel therapeutic target in CVID as well as other more common conditions characterized by sterile inflammation or autoimmunity.

## Introduction

Common variable immunodeficiency (CVID) is a heterogeneous disease where the immune system fails to produce sufficient amounts of antibodies, resulting in reduced levels of immunoglobulin (Ig)G, IgM and/or IgA. It is the most common symptomatic primary immunodeficiency in adults with a prevalence of 1:25 000–1:50 000 in Caucasians^1^. Monogenic causes are identified in approximately 10–20% of CVID patients^2–4^, but in most cases the etiology is unknown though might include polygenic variations in addition to epigenetic modifications^5,6^. The clinical hallmark of CVID is increased susceptibility to respiratory tract infections with encapsulated bacteria. Paradoxically, a large proportion of CVID patients also have autoimmune and non-infectious inflammatory complications such as lymphoid hyperplasia, granuloma, lymphoid interstitial pneumonitis, gastrointestinal inflammation and a variety of autoimmune disorders^7^. In line with this, CVID patients show signs of persistent systemic inflammation including monocyte/macrophage and T-cell activation, with increased levels of inflammatory mediators such as C-reactive protein (CRP), tumor necrosis factor (TNF), soluble (s) CD25 and sCD14^8–11^.

In view of the wide variety of phenotypes in CVID, it is useful to subdivide the patient group into two main categories: those with infections only and those who also display non-infectious complications. Importantly, CVID patients with one or more of these non-infectious complications have an 11-fold increase in mortality^7^. Whereas IgG replacement therapy reduces the severity and frequency of infections, it has little effect on the inflammatory and autoimmune complications^12^. It is therefore of major importance to elucidate the mechanisms of these non-infectious complications in order to identify new targets for therapy in CVID^13^.

The anti-inflammatory properties of high-density lipoprotein (HDL) are well established in atherosclerotic disorders, but have also been suggested in non-atherosclerotic conditions such as sepsis, autoimmune disorders and cancer^14–16^. *In vitro* and *in vivo* experiments have suggested an anti-inflammatory role for HDL by its ability to counteract toll-like receptor (TLR)-induced inflammation in macrophages via activating transcription factor 3 (AFT3)^17^. Decreased cholesterol efflux capacity from peripheral cells, which is influenced by HDL activity, has in murine models of dendritic cells been shown to result in autoimmunity and inflammation via increased intracellular inflammasome activity^18^.

Based on the above, we hypothesized HDL levels and functional activity to be altered in CVID, possibly contributing to systemic immune activation and inflammation as well as development of autoimmunity. We explored this hypothesis both by conducting analyses of lipid profiles in a large CVID population and by running experimental studies of HDL function in relation to cholesterol efflux capacity and anti-inflammatory effects in CVID patients and controls. Here, we present novel insights into the role of HDL in systemic inflammation of CVID, findings that can be of relevance to other autoimmune and inflammatory disorders as well.

## Results

### Patients’ characteristics

Clinical characteristics of the 102 CVID patients included in the lipid- and inflammatory analyses are shown in Table 1. In addition, a complete description of their autoimmune and inflammatory complications is given in Supplemental Table S1. Not all sub analyses and *in vitro* experiments were performed in all patients and so clinical characteristics of the 40 patients making up the smaller cohort, used for HDL subclass analyses and PBMC gene expression studies, and also the cohorts used for functional studies are shown in Supplemental Table S2. A complete overview of the CVID patients participating in the different analyses is given in Supplemental Table S3.

**Table 1:** Patient characteristics for CVID cohort and healthy controls.

	CVID cohort (n = 102)	Healthy controls (n = 28)	p-value
Age in years mean ± SD [min-max]	48 ± 15 [18–83]	42 ± 10 [28–65]	0.053^*^
Female (%)	54%	64%	0.328^†^
BMI mean ± SD [min-max]	25 ± 4 [17–39]	24 ± 3 [19–34]	0.232^‡^
IVIG (%)	18 (18%)	—	—
SCIG (%)	71 (70%)	—	—
IVIG and SCIG^’^ (%)	12 (12%)	—	—
Infections only (%)	26 (26%)	—	—
Non-infectious complications (%)	76 (75%)	—	—

BMI: Body mass index. IVIG: Intravenous immunoglobulins. SCIG: Subcutaneous immunoglobulins.

‘One CVID patient did not receive any immunoglobulin substitution.

*Mann Whitney test, ^†^Pearson Chi square test, ^‡^Student’s t-test.

### The CVID patients had decreased HDL cholesterol levels

**Figure 1:**
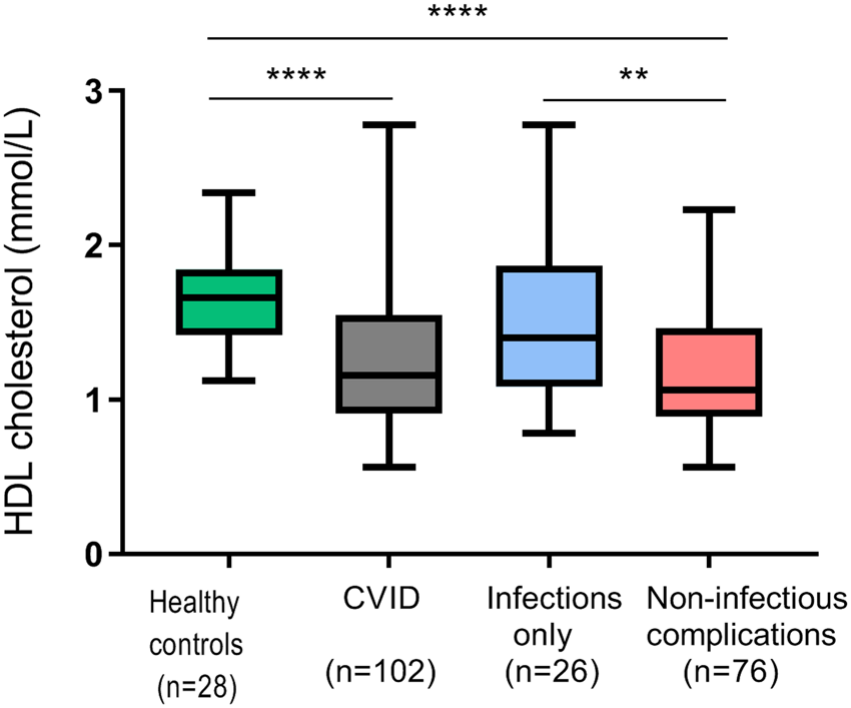
HDL cholesterol levels in CVID patients and controls including CVID subgroups. Plasma levels of HDL cholesterol in Common Variable Immunodeficiency (CVID) patients and controls. The CVID cohort is further divided into two subgroups: infection only and non-infectious complications. Results are given as boxes representing the 25^th^ to 75^th^ percentile with lines indicating median and whiskers min-max values; **p < 0.01, ****p < 0.0001 using Mann-Whitney test between groups.

We measured the total cholesterol, HDL cholesterol and LDL cholesterol levels in plasma from 102 CVID patients and 28 healthy controls. Compared to healthy controls, CVID patients had lower plasma HDL levels (1.66 [1.42, 1.84] vs 1.16 [0.91, 1.55], p < 0.0001, median [25^th^, 75^th^ percentile] mmol/L, respectively) (Fig. 1), whilst LDL (p = 0.99) and total cholesterol levels (p = 0.57) showed no significant difference. Low HDL levels in CVID patients compared to controls were consistent after correcting for sex, BMI and age (p < 0.001).

In 32 of the CVID patients we had two HDL measurements (mean time gap between sample collection 21 months, range 8–28 months) and found no significant difference between HDL levels measured at the two different time points (p = 0.42), suggesting that decreased HDL levels is a stable feature in CVID (Supplemental Fig. S1).

Out of 102 CVID patients, seven (7%) were using statins and 18 (18%) had a disease history of cardiovascular disease (defined as hypertension requiring medical treatment [n = 16] or coronary artery disease [n = 2]). Importantly, HDL cholesterol levels in these patients did not differ from the other CVID patients (1.12 ± 0.13 in statin users vs 1.26 ± 0.04 in non-statin users; p = 0.439, and 1.15 ± 0.11 for patients with cardiovascular disease vs 1.28 ± 0.05 for those without cardiovascular disease; p = 0.234, HDL levels given in mean ± SEM mmol/L).

### Low HDL cholesterol levels were associated with systemic inflammation and non-infectious complications in CVID

When dividing the CVID cohort into subgroups by phenotype, we found significantly lower HDL levels in CVID patients with non-infectious complications (n = 76) than in CVID patients with infections only (n = 26) (1.1 [0.9, 1.5] vs 1.4 [1.1, 1.9] respectively, p < 0.01, HDL results given as median [25^th^, 75^th^ percentile] mmol/L) (Fig. 1). There was no significant difference between healthy controls and patients with infections only (p = 0.09), hence the low HDL levels in those with non-infectious complications were driving the difference between healthy controls and CVID patients overall (p < 0.0001).

**Figure 2:**
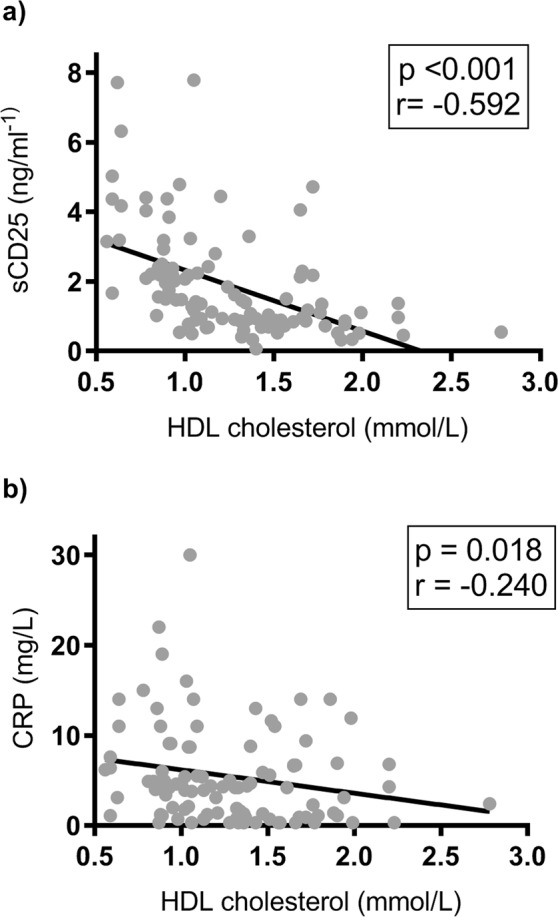
Correlation between HDL cholesterol levels and inflammatory markers in CVID. Panels show correlation between HDL cholesterol and sCD25 **(a)** and CRP **(b)** in CVID patients (n = 102). Comparisons are made by Spearman’s rank correlation test. Spearman’s rank correlation coefficient is referred to as r. Trend lines indicate negative correlation between variables sCD25 and HDL cholesterol as well as between CRP and HDL cholesterol.

Furthermore, we found a negative correlation between plasma levels of HDL cholesterol and sCD25 (r = −0.59, p < 0.001) and CRP (r = −0.24, p = 0.018) (Fig. 2a,b), but not with LPS (r = −0.08, p = 0.407) or sCD14 (r = −0.16, p = 0.119) in CVID patients. Combined, these findings suggest that the decreased levels of HDL cholesterol in CVID are associated with increased systemic immune activation and an inflammatory and autoimmune phenotype. Stepwise regression identified sCD25 (beta = −0.52, p = 0.001) and sex (beta = −0.33, p = 0.034) as the strongest predictors of HDL cholesterol levels in CVID (Supplemental Table S4).

### HDL subclasses and Apo A-1 in CVID

**Figure 3:**
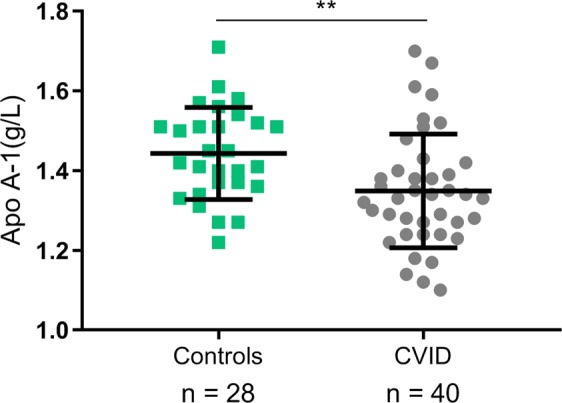
Apo A-1 levels in CVID patients and controls. The results are given as mean with error bars for SD. **p < 0.01 using unpaired Student’s t-test between groups.

HDL particles are heterogeneous and can be fractioned into subclasses defined by density or size reflecting differences in relative content of proteins and lipids: small (S), medium (M), large (L) and extra-large (XL). Hence, the HDL subclasses vary in composition and also differ in capacity of contributing to reverse cholesterol transport as well as anti-oxidative and anti-inflammatory activity^19^. Several studies have found lipid-free Apo A-1 and the smaller forms of HDL to be the main acceptors of cholesterol efflux via the ATP-binding cassette transporter (ABCA1) pathway in macrophages^20,21^. We therefore next measured plasma concentration of Apo A-1 and each HDL subclass in 40 CVID patients and 28 healthy controls (characteristics are given in Supplemental Table S2). CVID patients had significantly lower levels of M-HDL (p = 0.025), L-HDL (p = 0.005) and XL-HDL (p = 0.041), but not S-HDL (p = 0.778) (Supplemental Fig. S2). Importantly, Apo A-1, the main protein and functional component of HDL^19,22^ was also significantly lower in our CVID patients compared to healthy controls (p = 0.005) (Fig. 3). Thus, although the normal proportion of S-HDL could suggest a normal reverse cholesterol transport (RCT) function, HDL particles in CVID had markedly reduced levels of Apo A-1, predicting impairment of function.

### Serum from CVID patients had decreased cholesterol acceptor function

**Figure 4:**
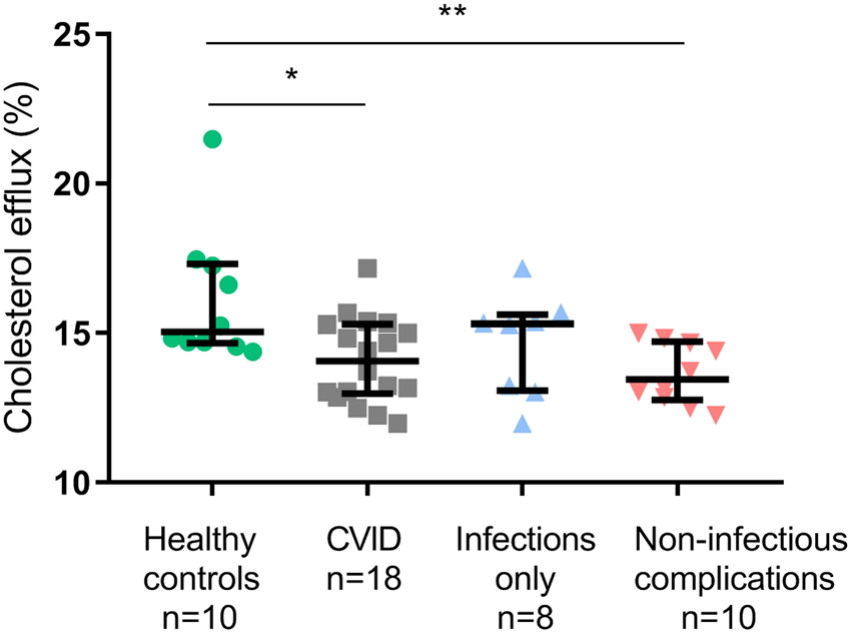
Cholesterol efflux from THP-1 macrophages to serum in CVID patients and controls. The results are given as median with interquartile range showing cholesterol efflux in CVID patients and healthy controls as well as in the CVID subgroups: infection only and non-infectious complications. *p < 0.05, **p < 0.01 using Mann-Whitney test between groups.

To further look into the functional capacity of HDL in CVID patients, we tested the cholesterol acceptor function of HDL in a subgroup of CVID patients (n = 18) and healthy controls (n = 10); characteristics are given in Supplemental Table S2. Serum was employed, as its cholesterol acceptance is mainly driven by HDL function and concentration. We exposed THP-1 macrophages to ^14^C-cholesterol for  48 hours before cholesterol efflux was induced by serum from either CVID patients or controls. We found significantly decreased cholesterol acceptor capacity in serum from CVID patients compared to healthy controls (14.1% [13.0%, 15.3%] versus 15.0% [14.7%, 17.3%], p < 0.05, results given as median [25% percentile, 75% percentile]) (Fig. 4), illuminating reduced function of a key step in RCT in these patients. Furthermore, when dividing the patients into the two clinical subgroups, serum from CVID patients with non-infectious complications had significantly reduced cholesterol acceptor capacity (13.5% [12.7%, 14.7%]) compared to controls (p = 0.003), in contrast with those who suffered from infections only (15.3% [13.1%, 15.6%], p = 0.46, all results given as median [25% percentile, 75% percentile]) (Fig. 4). These findings suggest that CVID patients with autoimmune and inflammatory complications have decreased HDL function and not only decreased HDL levels.

### CVID patients had decreased expression of ABCA1

**Figure 5:**
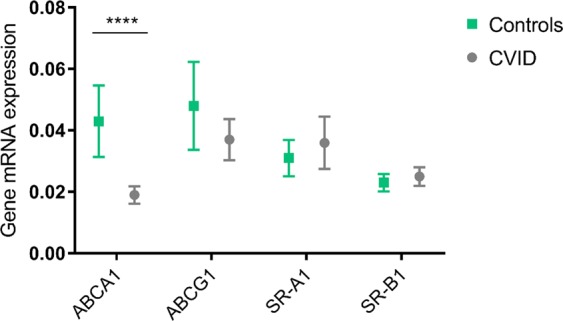
Reverse cholesterol transport related gene expression in PBMC from CVID patients and controls. mRNA expression in PBMC from CVID patients (n = 40) and healthy controls (n = 30) of the genes: ABCA1 (****p < 0.0001), ABCG1 (p = 0.17), SR-A1 (p = 0.55) and SR-B1 (p = 0.32) involved in reverse cholesterol transport from peripheral cells, using Mann-Whitney test between groups. Results given as mean with 95% CI. mRNA levels were quantified by qPCR and values given in relation to the reference genes β-actin and GAPDH.

Reversed cholesterol transport is not only dependent on HDL acceptor capacity, but also the cellular transport properties of the cholesterol-loaded cells. Further exploring cellular transport activity, we analyzed mRNA expression of RCT related genes measured by qPCR on PBMC from 40 CVID patients and 30 healthy controls (characteristics are given in Supplemental Table S2). We found significantly lower levels of ABCA1 mRNA in CVID patients than in healthy controls (p < 0.0001) (Fig. 5). There was no significant difference in mRNA expression of ABCG1 (p = 0.17), SR-A1 (p = 0.55) or SR-B1 (p = 0.32) between CVID patients and healthy controls, respectively (Fig. 5).

### Cholesterol efflux capacity decreased in macrophages from CVID patients

**Figure 6:**
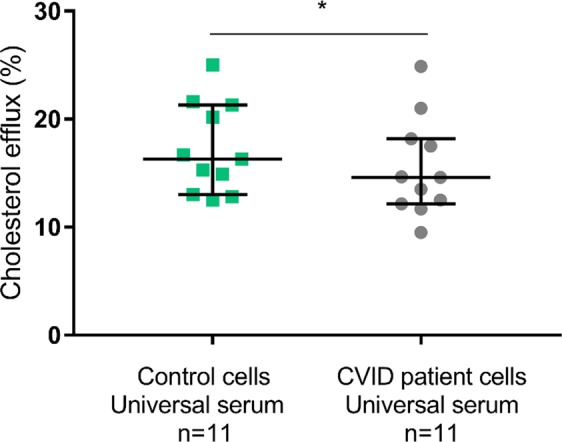
Cholesterol efflux from CVID and control macrophages to universal serum. Cholesterol efflux from monocyte-derived macrophages from CVID patients with non-infectious complications and age- and sex matched healthy controls to universal serum. Results given with bars for median and interquartile range, *p < 0.05 using Wilcoxon matched pair signed rank test.

In order to further assess reverse cholesterol transport capacity in CVID, we used monocyte derived macrophages from 11 CVID patients and 11 age and sex matched controls (in this particular experiment only CVID patients with non-infectious complications were included; Supplemental Table S2) and a uniform control serum (2.5%, v/v) as acceptor. We exposed macrophages to ^14^C-cholesterol for  48 hours for cholesterol loading followed by overnight stabilization in medium without additives before efflux was analyzed. Whereas there was no difference in cholesterol loading between macrophages from CVID patients and controls (data not shown), macrophages from CVID patients had significantly reduced cholesterol efflux capacity as compared to macrophages from healthy controls (14.6% [12.2%, 18.2%] vs 16.3% [13.0%, 21.3%], p = 0.04, results given as median [25% percentile, 75% percentile]) (Fig. 6), consistent with our findings of low ABCA1 expression in CVID patients.

### Impaired anti-inflammatory effect of HDL on TLR stimulation of PBMC from CVID patients

**Figure 7:**
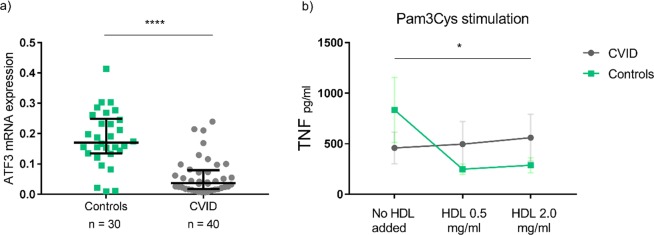
ATF3 mRNA levels and HDL cholesterol effects on TLR2-stimulated TNF release from mononuclear cells in CVID patients and controls. (**a**) ATF3 mRNA expression in CVID patients and healthy controls; results given with bars for median and interquartile range, ****p < 0.0001 using Mann-Whitney test between groups. mRNA levels were quantified by qPCR and values given in relation to the reference genes β-actin and GAPDH. (**b**) Effect of increasing HDL cholesterol concentration on TLR2- (Pam3Cys) stimulated TNF release from mononuclear cells in CVID (n = 6) and controls (n = 6); *p < 0.05. Results illustrated as median with variance, p-values calculated using repeated measures ANOVA analysis. The difference between CVID patients and the control group in TNF release at baseline from mononuclear cells after Pam3Cys stimulation was found non-significant (p = 0.389) when performing Student’s t-test on log-transformed datasets.

HDL has recently been shown to attenuate TLR mediated inflammatory response in macrophages as assessed by IL-6 and TNF measurements, at least partly mediated by the transcriptional regulator ATF3^17^. Thus, we next examined the interaction between HDL and ATF3 in PBMC from CVID patients and controls. PBMC from 40 CVID patients had decreased ATF3 mRNA levels compared to PBMC from 30 healthy controls (p < 0.0001, Fig. 7a) (cohort characteristics given in Supplemental Table S2). We explored this further by incubating freshly isolated PBMC from six CVID patients and six age and sex matched healthy controls (in this particular sub-study only CVID patients with an inflammatory phenotype were included; Supplemental Table S2) in the presence of different concentrations of human HDL for 6 hours. We then stimulated the cells with TLR4 (LPS) and TLR2 (Pam3Cys) ligands for 12 hours, and found a suppressive effect of HDL on TNF release in Pam3Cys exposed cells in healthy controls (Fig. 7b). Notably, however, the suppressive effect of HDL on TNF release was significantly decreased in cells from CVID patients stimulated with TLR2 ligand Pam3Cys (Fig. 7b), but not with TLR4 ligand LPS. The same effect was not observed for IL-6 release (Supplemental Fig. S3).

## Discussion

In the present study, we show decreased levels of HDL cholesterol associated with systemic inflammation and inflammatory and autoimmune complications in CVID. Our functional studies demonstrate impaired HDL cholesterol acceptor function and impaired reverse cholesterol transport from macrophages in CVID, likely related to decreased levels of Apo A-1 and low mRNA expression of ABCA1 in these patients. Furthermore, our findings suggest attenuated anti-inflammatory effects of HDL on PBMC from CVID patients, which could reflect their decreased ATF3 expression. This study proposes new pathogenic mechanisms for the clinically significant systemic immune activation and non-infectious complications in CVID, expanding the role of HDL as an important modulator of inflammation.

One study has previously shown reduced HDL cholesterol and Apo A-1 levels in 18 patients with CVID and six patients with X-linked agammaglobulinaemia compared to 12 healthy controls, with a negative correlation between plasma levels of HDL and TNF in these patients^23^. Herein we extend these findings by showing decreased HDL cholesterol levels in a substantially larger study population, and further demonstrate that the reduced HDL cholesterol levels are stable during temporal testing. Importantly, the lowest HDL levels were seen in CVID patients with autoimmune and inflammatory complications. This suggests that low HDL levels are related to sterile inflammation rather than infectious-driven inflammation, supported by the detection of sCD25, a robust marker of T cell activation, as the strongest predictor for low HDL levels. In line with our findings, previous studies have found low HDL levels associated with disease activity and systemic inflammation in autoimmune diseases such as inflammatory bowel disease, rheumatoid arthritis, systemic lupus erythematosus and Sjogren’s syndrome^24–27^.

The detailed study of HDL particle composition gave some discordant results showing normal proportions of the small HDL particles but decreased levels of Apo A-1 in CVID, both particles of major importance to HDL function. However, our studies using control and patient serum to reflect HDL acceptor capacity for cholesterol in RCT showed significantly impaired HDL function in CVID, primarily reflecting impaired cholesterol acceptor capacity in CVID patients with non-infectious complications. The consequence of this impaired HDL acceptor function was augmented by decreased cholesterol efflux from macrophages in CVID patients, likely related to decreased expression of the ATP-binding cassette transporter ABCA1.

A major finding in the present study was the association between decreased levels and function of HDL and systemic inflammation. These correlations could also be supported by functional studies. The combined impairment of cholesterol acceptor function in serum and reduced reverse cholesterol transport from macrophages likely leads to lipid accumulation in these cells. A similar pattern in dendritic cells has been demonstrated to promote a TLR mediated inflammatory response^28^. Additionally, we found that PBMC from CVID patients had lower expression of transcription factor ATF3 mRNA along with a modestly attenuated anti-inflammatory effect of HDL as assessed by TNF release in TLR2 activated PBMC. ATF3 has been found to be vital in the protective effect of HDL against TLR induced inflammation^17^. Increased TNF levels are typical of the inflammatory CVID phenotype^8^, and our findings could relate this phenotype to impaired cellular HDL responses.

A rational line of reasoning could suggest the chronic systemic inflammation found in CVID patients would lead to increased incidence of cardiovascular disease in this group. However, no clear evidence has been found to substantiate this hypothesis^29^. American and European registry data have rather identified lung disease and autoimmune manifestations as the most prevalent complications to CVID^7,30^. It has however been speculated that atherosclerosis is present but does not amount to clinical levels substantial enough to cause cardiovascular disease due to the reduced life expectancy in CVID patients with non-infectious complications^29^.

The reasons for the decreased HDL levels and function in CVID patients are, at present, not clear. We have previously shown GI tract inflammation in a large proportion of CVID patients^31^ which could influence lipid metabolism. Patients with CVID enteropathy have been found to have low mucosal IgA levels in the duodenum, which has been suggested could shift intestinal molecular pathways from appropriate lipid metabolism to enteropathy-inducing immune processes^32^. The gut microbiota has previously been shown to contribute to variation in blood lipids^33^ and alteration of the gut microbiota composition in CVID has been linked to systemic inflammation^34^. Importantly, whereas inflammation *per se* could have contributed to the decreased HDL levels^35^, decreased HDL levels may further increase inflammation in CVID patients, hence representing a pathogenic loop. In a clinical setting, such a loop could be targeted therapeutically by Apo A-1 mimetics^36^, cholesteryl transfer protein (CETP)-inhibitors^37^, statins or a combination thereof.

Based on the prevalence of CVID, the total number of patients in the cholesterol analysis part of the study was large and the study included robust characteristics of HDL composition and function, viewed as a major strength of this study. Still there are some limitations, including a rather low number of patients in some of the functional sub-studies. Furthermore, the blood samples were not obtained at a fasted state. We do however suggest that this should only have minor influences on the HDL data. Finally, we lack protein data on some important molecules like ABCA1 and ATF3, which could have been useful as mRNA may not necessarily reflect the protein expression of these molecules.

In conclusion, the present study shows novel data demonstrating marked disturbances in HDL cholesterol levels and HDL function in CVID, with HDL levels inversely correlating with inflammatory markers and a more severe phenotype in these patients. Furthermore, it shows reduced HDL function via impaired reverse cholesterol transport in CVID patients, which appears to be an important factor contributing to their systemic inflammation and autoimmune and inflammatory manifestations. The identification of HDL function as a target for treatment to reduce inflammatory and autoimmune complications in CVID should be further explored. Our findings support a link between a dysregulated immune system, lipid metabolism, inflammation and autoimmunity in humans. Indeed, the findings of this study could be transferable and therapeutically useful to other and more common inflammatory and autoimmune diseases.

## Methods

### Study population

CVID patients were recruited from the outpatient clinic at the Section of Clinical Immunology and Infectious Diseases, Oslo University Hospital Rikshospitalet, which functions as a national center for diagnosis and treatment of adults with primary immunodeficiency diseases in Norway. CVID was defined as decreased serum levels of IgG, IgA and/or IgM by a minimum of two standard deviations below the mean for age, whilst excluding other causes of hypogammaglobulinemia. Patients with ongoing acute infections and patients using immunosuppressive drugs were excluded, whereas use of statins was not an exclusion criterion. The larger cohort of 102 CVID patients was recruited from November 2011 to December 2012 and the smaller cohort of 40 CVID patients was recruited between October 2013 and October 2014 as previously described^34,38^ (Supplemental Fig. S4). For comparison, we included 30 healthy controls based on disease history and on no regular medications. Blood was drawn for PBMC isolation, serum and plasma samples from all controls. However, technical issues with the plasma samples from two healthy controls lead to a reduced number of controls (n = 28) for the plasma analyses. Supplemental Table S3 gives a detailed overview of which CVID patients contributed to the different functional studies and which were also part of the large and small cohorts. For cellular studies on macrophage cholesterol efflux and the effect of HDL on TLR-stimulated release of TNF and IL-6 from macrophages, we specifically wanted to focus on the possible pathological mechanisms in patients with systemic inflammation and autoimmunity and therefore selected CVID patients with known non-infectious complications for these particular analyses.

### Blood sampling

Plasma for lipid analyses was sampled into EDTA collection tubes, immediately immersed in melting ice and centrifuged within 30 minutes at 2,500 *g* for 20 minutes prior to being stored at −80 °C until analysis. For patients on intravenous Ig therapy, blood samples were collected just prior to infusion.

### Analyses of lipid and inflammatory parameters

Total cholesterol, HDL cholesterol, Apo A-1 and low density lipoprotein (LDL) cholesterol were measured enzymatically on a Hitachi 917 system (Roche Diagnosis GmbH, Mannheim, Germany) using the cholesterol (cholesterol CHOD-PAP), HDL-cholesterol plus (catalog no. 04713257190) and LDL-cholesterol plus (catalog no. 04714423190) kits from Roche Diagnostics. CRP levels were analysed via the routine hospital laboratory on the day of sampling using a high-sensitivity method. Plasma levels of sCD14 and sCD25 were quantified in duplicate by enzyme immunoassays obtained from R&D Systems (Minneapolis, MN). Endotoxin was analysed by Limulus Amebocyte Lysate chromogenic assay (Lonza, Walkersville, MD) according to the manufacturer’s instructions, with the following modifications: samples were diluted 10-fold to avoid interference with background colour and preheated to 68 °C for 10 minutes prior to analysis to dissolve immune complexes. Supernatant TNF and IL-6 levels were analyzed with a V-plex Proinflammatory Panel 1 kit from Meso Scale Discovery (Meso Scale Diagnostics, LLC, 1601 Research Blvd. Rockville, MD) using QuickPlex SQ120.

### Characterizing HDL sub-fractions

EDTA plasma samples were quantified using a commercial high-throughput proton NMR metabolomics platform (Nightingale Health Ltd, Helsinki, Finland). Details of the experimentation and applications of the NMR metabolomics platform have previously been described^39^. The mean size for the HDL particles was calculated by weighting the corresponding subclass diameters with their particle concentrations, and the four HDL subclass sizes were defined as 14.3 nm (XL), 12.1 nm (L), 10.9 nm (M), and 8.7 nm (S).

### Isolation of PBMC

Peripheral blood mononuclear cells (PBMC) were isolated from heparinised venous blood by gradient centrifugation using Lymphoprep (Axis Shield, Oslo, Norway) within 1 hour after blood collection. PBMC were used for further *in vitro* experiments immediately following isolation or stored as pellet in −80 °C for PCR analyses.

### Quantitative real-time (RT)-PCR analyses

Total RNA was isolated from PBMC using RNeasy spin columns as described by the manufacturer (Qiagen, Hilden, Germany). Isolated RNA was treated with DNase (Qiagen) and stored at −80 °C for later analysis. RNA concentrations and purity were assessed by spectrophotometer absorbance (NanoDrop ND-1000 Thermo Scientific, Wilmington, DE). 500 ng of RNA was loaded into the cDNA synthesis using q-Script cDNA Synthesis kit (Quanta Bioscience, Gaithersburg, MD). Quantification of mRNA was performed using Perfecta SYBR Green qPCR Fast Mastermix (Quanta Bioscience) on the 7900HT Fast Real-Time PCR System (Applied Biosystems, Foster City, CA) with the accompanying software SDS 2.4. All primer sequences can be provided upon request. As small volumes were used in the analyses of human samples, robot pipetting for RT-qPCR reactions was employed. For each transcript, RT-qPCR was conducted in duplicate. Target transcript levels were quantified by the comparative Ct method using the average Ct-median value from reference genes β-actin and GAPDH as endogenous control.

### Functional studies

#### Serum as cholesterol acceptor

The human monocytic cell line THP-1 monocytes (ATCC, Chicago, IL) were differentiated to THP-1 macrophages using 12-myristate 13-acetate (PMA, Sigma) (100 nM). After 24 hours the macrophages were loaded with ^14^C-cholesterol (0.5 µCi/ml [18.5 mBq/l] American Radiolabel Chemicals, Saint Louis, MO) in regular growth medium added oxidized LDL (10 µg/ml) and further grown for 48 hours. After overnight incubation in sterile 0.2% (wt/v) human serum albumin in RPMI1640, the cholesterol acceptor capacity was measured adding 2.5% (v/v) heat-inactivated serum (in RPMI-1640 medium) from CVID patients and healthy controls to the lipid laden THP-1 macrophages. After 3 hours incubation, the medium was collected and ^14^C-cholesterol measured by liquid scintillation counter (TRI-CARB 2300 TR Scintillation Counter [Packard, Waltham, MA]). Total cell-associated ^14^C-cholesterol loading of THP-1 macrophages was assessed in a parallel set up. Percentage efflux was calculated using the equation: [DPM_medium_/(DPM _(cell+medium)_)] × 100.

#### Cholesterol efflux capacity

Cell viability in the PBMC suspensions was tested using Countess^TM^ automated cell counter, before seeding onto a 24-well plate (Nunclon^TM^ Delta Surface, Thermo Fisher scientific, MA). The cells were incubated for an hour, washed twice, and plastic adhered monocytes were further stimulated with TNF 10 ng/ml (R&D Systems) for the mononuclear cells to differentiate into a macrophage-like phenotype. After two days, the macrophage-like cells were loaded with ^14^C-cholesterol (0.5 µCi/ml [18.5 mBq/l]) in regular growth medium added oxidized LDL (10 µg/ml). After 48 hours lipid loading, the cells were incubated overnight with 0.2% (wt/v) human serum albumin in RPMI1640. The patient and control cells’ efflux capacity were assessed using 2.5% heat inactivated serum from one healthy individual as detailed above.

#### ATF3 and anti-inflammatory reprogramming

PBMC were isolated as described above. The cells were pre-treated for 6 hours with HDL (0.5 mg/ml, 2 mg/ml and no HDL, respectively [purified human HDL, Kalen Biomedical, LLC, Germantown, MD]). They were then stimulated overnight with the TLR4 ligand LPS from *Escherichia coli* 026:B6 (2.5 ng/ml) and TLR2 ligand Pam3Cys (0.8 µg/ml), before supernatant levels of TNF and IL-6 were measured as described above.

### Statistics

The datasets for HDL analyses, including HDL subclasses, were not normally distributed as per Kolmogorov-Smirnov normality tests and the data was therefore log transformed. Where normal distribution was achieved, we continued with two-tailed multivariate testing. Where normal distribution was not achieved, we applied Mann-Whitney non-parametric testing, all tests with significance level 0.05. We ran bivariate correlation analyses in addition to forced and stepwise linear regression analyses to investigate the connections between lipid levels and inflammatory markers. For the longitudinal data, we compared two different time points using related samples Wilcoxon signed rank test. For the functional studies with age and sex matched controls we applied matched pair Wilcoxon signed rank test, and where repeated measurements were done under various HDL concentrations we used repeated measures ANOVA analysis.

### Study approval

The study was approved by the Regional Committee for Medical and Health Research Ethics of South-Eastern Norway (number 2012/521, 2013/1037) and conducted in accordance with the Helsinki Declaration. All participants provided written, informed consent. The study participants were given a study number which was used for handling samples and analyses throughout the study.

## Supplementary information

Supplemental material

## Data Availability

The datasets generated and analysed during the current study are available from the corresponding author on reasonable request.
