# Supplementary material for: Impaired HDL Function Amplifies Systemic Inflammation in Common Variable Immunodeficiency
Source: Sci Rep. 2019 Jul 1;9:9427. doi: 10.1038/s41598-019-45861-1 (PMC6603020; doi:10.1038/s41598-019-45861-1)
Supplement: Supplementary file 1 — Supplemental material [file 41598_2019_45861_MOESM1_ESM.pdf]

SUPPLEMENTAL MATERIAL

IMPAIRED HDL FUNCTION AMPLIFIES SYSTEMIC INFLAMMATION IN COMMON VARIABLE IMMUNODEFICIENCY

Magnhild E. Macpherson*, Bente Halvorsen, Arne Yndestad, Thor Ueland, Tom E. Mollnes, Rolf K. Berge, Azita Rashidi, Kari Otterdal, Ida Gregersen, Xiang Y. Kong, Kirsten B. Holven, Pål Aukrust, Børre Fevang, Silje F. Jørgensen.

*Corresponding author: Magnhild E. Macpherson, Research Institute of Internal Medicine, Oslo University Hospital, Rikshospitalet, P.Box 4950 Nydalen, 0424 Oslo, Norway. E-mail: m.e.macpherson@studmed.uio.no Telephone: +47 23070000

Table of content

Supplemental figure S1

Supplemental figure S2

Supplemental figure S3

Supplemental figure S4

Supplemental table S1

Supplemental table S2

Supplemental table S3

Supplemental table S4

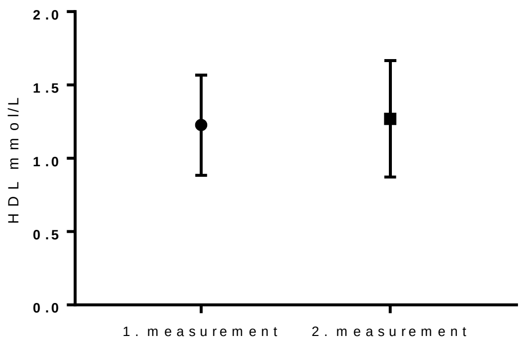

Supplemental Figure S1: HDL measurements stable over time in CVID patients

Longitudinal data for HDL in CVID replication cohort (n=32). p=0.42 using Wilcoxon matched-pair signed rank test. Data presented as mean with SD.

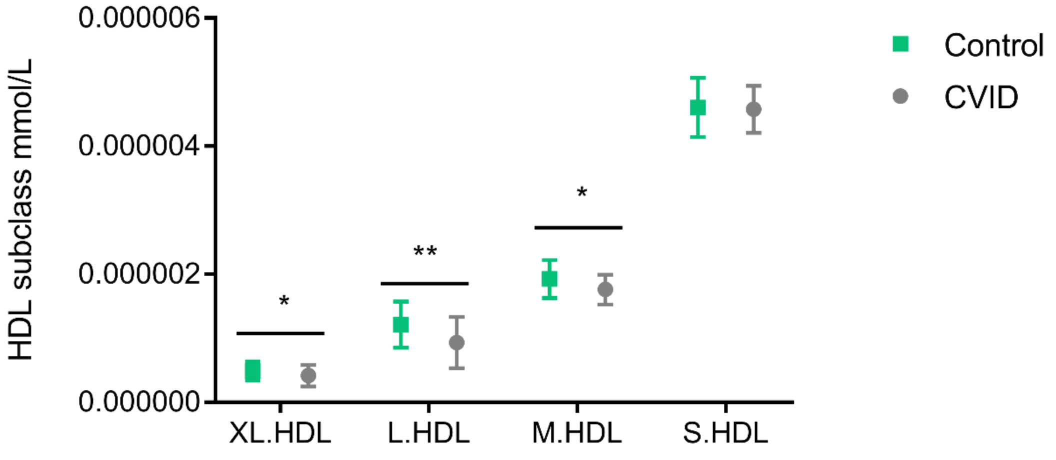

Supplemental figure S2: HDL subclasses in CVID patients and controls

CVID patients (n=40) have significantly lower levels of XL.HDL (*p<0.05), L.HDL (**p<0.01) and M.HDL (*p<0.05) than healthy controls (n=28), p-values calculated using Mann-Whitney test between groups. They also have lower levels of S.HDL than controls, but this difference does not reach statistical significance. Data presented as mean with SD.

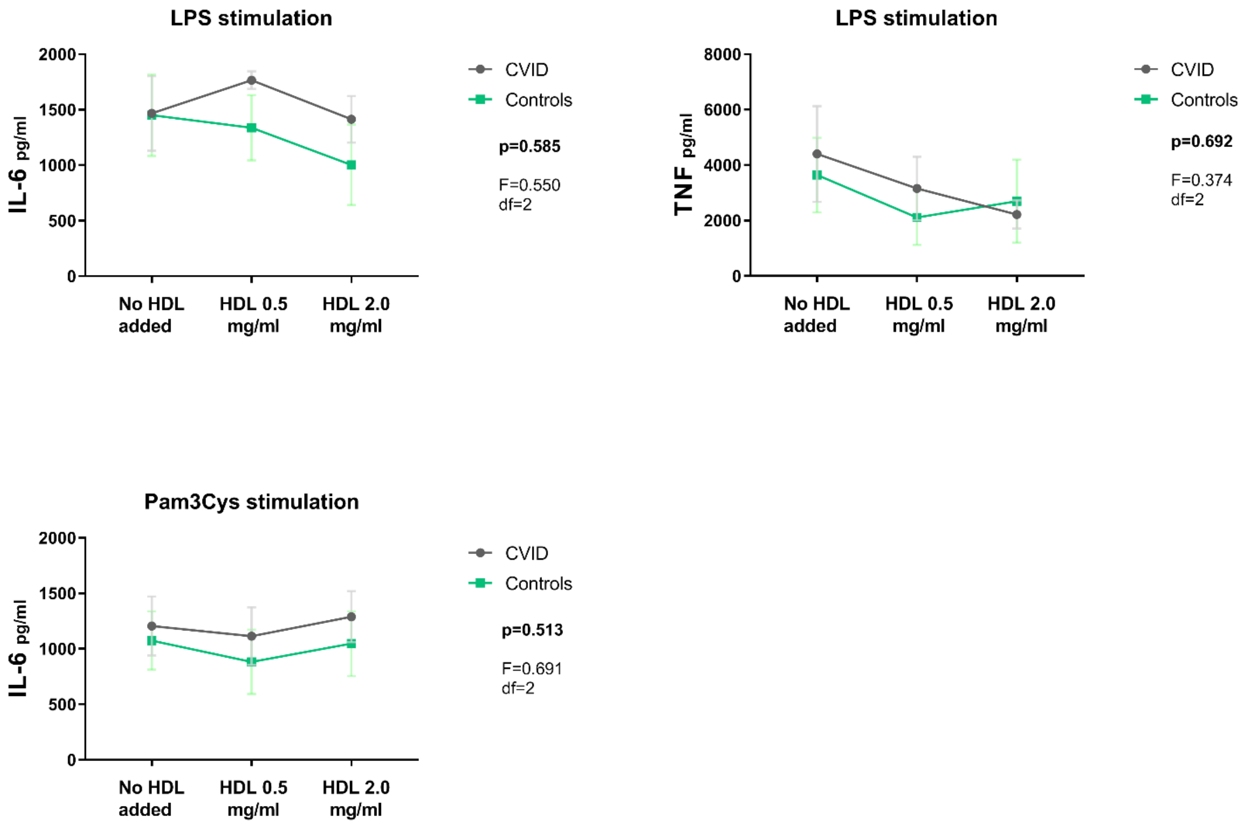

Supplemental Figure S3: HDL effects on TLR4-stimulated TNF and IL-6 release and TLR2-stimulated IL-6 release from mononuclear cells from CVID patients (n=6) and healthy controls (n=6)

Results given as median with variance, p-values calculated using repeated measures ANOVA analysis.

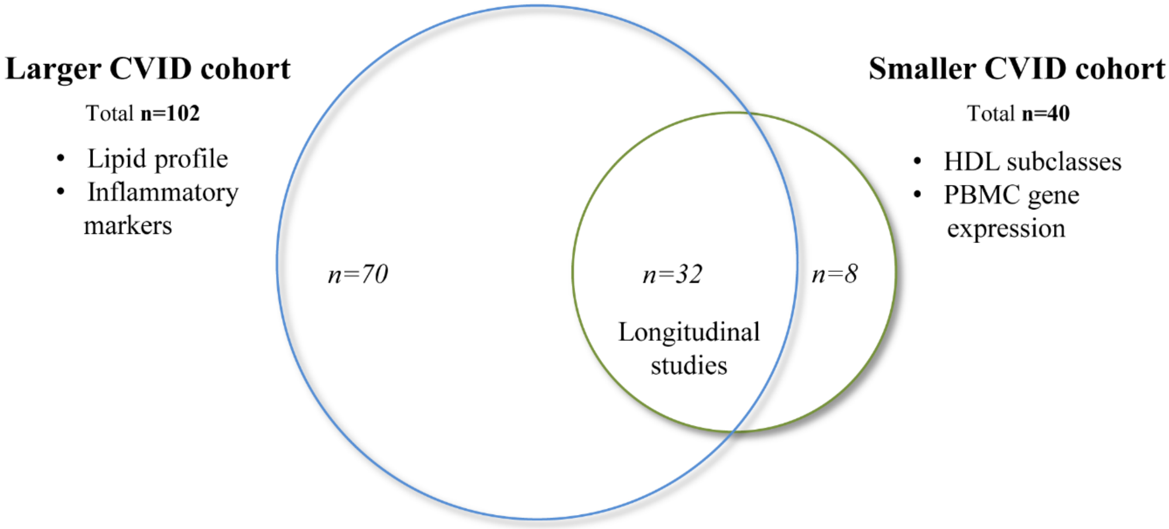

Supplemental Figure S4: Larger and smaller CVID patient cohorts

The larger CVID patient cohort (n=102) was collected between November 2011 and December 2012 and the smaller CVID cohort (n=40) was collected between October 2013 and October 2014. For both cohorts the patients were recruited from the outpatient clinic at the Section of Clinical Immunology and Infectious Diseases at Oslo University Hospital Rikshospitalet. The cohorts were somewhat overlapping, allowing for longitudinal studies of HDL-levels over a range of 8-28 months in these 32 patients. In the larger cohort, 74.5% had non-infectious complications, whereas in the smaller cohort 80% of the patients had non-infectious complications.

Supplemental table S1: Phenotype characteristics for CVID cohort

Phenotype	Complications to CVID (n=102) Number (%)
**Infection only[A]**	26 (25.5%)
**Non-infectious complications[B]**	76 (74.5%)
Splenomegaly	47 (46.1%)
Enteropathy	30 (29.4%)
Granulomas	15 (14.7%)
Organspecific autoimmunity	24 (23.5%)
Autoimmune Cytopenias	22 (21.6%)
Lymphoid Hyperplasia	48 (47.1%)
Lymphoma	1 (1.0%)
Nodular regenerative hyperplasia, liver	4 (3.9%)
Lymphocytic interstitial pneumonia	1 (1.0%)

[A]Infection only; recurrent bacterial airway infections as only clinical manifestation of CVID. [B]Non-infectious complications; exhibit one or more of the below listed autoimmune and inflammatory complications of CVID in addition to recurrent bacterial airway infections. CVID: common variable immunodeficiency.

Supplemental Table S2: Overview of CVID patients and healthy controls contributing to the different analyses and functional studies

HDL subclass analyses

	CVID patients (n=40)	Controls (n=28)	p-value
**Age** mean ± SD [min-max]	48 ± 12 [18-67]	42 ± 10 [28-65]	0.032[A]
**Female** number (%)	25 (63%)	18 (64%)	0.881[B]
**BMI** mean ± SD [min-max]	26 ± 5 [17-38]	24 ± 3 [19-34]	0.077[C]
**Non-infectious complications** (%)	80%	-	N/A

Gene expression analyses

	CVID patients (n=40)	Controls (n=30)	p-value
**Age** mean ± SD [min-max]	48 ± 12 [18-67]	43 ± 11 [28-65]	0.083[A]
**Female** number (%)	25 (63%)	18 (60%)	0.832[B]
**BMI** mean ± SD [min-max]	26 ± 5 [17-38]	24 ± 3 [19-34]	0.047[C]
**Non-infectious complications** (%)	80%	-	N/A

Serum cholesterol acceptor studies

	CVID patients (n=18)	Controls (n=10)	p-value
**Age** mean ± SD [min-max]	46 ± 10 [18-63]	43 ± 11 [28-64]	0.474[A]
**Female** number (%)	10 (56%)	6 (60%)	0.820[B]
**BMI** mean ± SD [min-max]	26 ± 5 [17-37]	24 ± 2 [22-27]	0.439[A]
**Non-infectious complications** (%)	56%	-	N/A

Macrophage cholesterol efflux studies

	CVID patients (n=11)	Controls (n=11)	p-value
**Age** mean ± SD [min-max]	39 ± 10 [22-54]	41 ± 9 [29-59]	0.717[A]
**Female** number (%)	9 (82%)	9 (82%)	1.000[B]
**BMI** mean ± SD [min-max]	26 ± 6 [19-39]	22 ± 2 [19-27]	0.081[A]
**Non-infectious complications** (%)	100%	-	N/A

HDL effects on TLR-stimulated IL-6 and TNF release in macrophages

	CVID patients (n=6)	Controls (n=6)	p-value
**Age** mean ± SD [min-max]	40 ± 9 [31-53]	41 ± 7 [33-49]	0.914[A]
**Female** number (%)	5 (83%)	5 (83%)	1.000[B]
**BMI** mean ± SD [min-max]	23 ± 5 [19-33]	22 ± 2 [19-25]	1.000[C]
**Non-infectious complications** (%)	100%	-	N/A

[A]Student's t-test, [B]Pearson Chi square test, [C]Mann Whitney test.

CVID: common variable immunodeficiency. BMI: Body mass index.

Supplemental Table S3: Overview of CVID patients contributing to the different cohorts

Patient	Larger cohort (n=102)	Smaller cohort (n=40)	Serum cholesterol acceptor study (n=18)	Cholesterol efflux from macrophages study (n=11)	HDL level, cytokine release study (n=6)
1-14	X	X	X		
15	X	X		X	X
16	X	X		X	
17	X	X			X
18	X			X	X
19	X			X	
20	X			X	
21-36	X	X			
37-102	X				
103		X			
104		X			
105		X	X		
106		X	X		
107		X	X		
108		X	X		
109		X		X	
110				X	
111				X	
112				X	
113					X
114					X
115					X
116				X	
117				X	

Supplemental Table S4: Stepwise regression analysis of variables that could predict HDL levels in the CVID cohort (n=102)

Variable	Beta coefficient	p-value
**sCD25**	-0.52	0.001*
**Sex**	-0.33	0.034*
**BMI**	-0.21	0.154
**CRP**	-0.21	0.194
**LPS**	-0.19	0.226
**Age**	0.13	0.413
**Smoking**	-0.10	0.515
**sCD14**	0.10	0.583

*p-value <0.05. Soluble CD25 and sex are the strongest predictors of HDL levels.

CVID: common variable immunodeficiency. BMI: Body mass index. sCD25: soluble CD25. CRP: C-reactive protein. sCD14: soluble CD14.
